# Supplementary material for: Multi‐dataset identification of innovative feature genes and molecular mechanisms in keratoconus
Source: J Cell Mol Med. 2024 Sep 19;28(18):e70079. doi: 10.1111/jcmm.70079 (PMC11412914; doi:10.1111/jcmm.70079)
Supplement: Supplementary file 2 — Material S2. [file JCMM-28-e70079-s002.zip › 7.GSEA analysis/2.ATOH7/KEGG_VIRAL_MYOCARDITIS.html]

Details for gene set KEGG\_VIRAL\_MYOCARDITIS[GSEA]

|  || Dataset | ATOH7.group.cls#h\_versus\_l |
| Phenotype | group.cls#h\_versus\_l |
| Upregulated in class | h |
| GeneSet | KEGG\_VIRAL\_MYOCARDITIS |
| Enrichment Score (ES) | 0.45327488 |
| Normalized Enrichment Score (NES) | 1.4205114 |
| Nominal p-value | 0.07321773 |
| FDR q-value | 0.29044053 |
| FWER p-Value | 0.885 |
Table: GSEA Results Summary

  

Fig 1: Enrichment plot: KEGG\_VIRAL\_MYOCARDITIS      
 Profile of the Running ES Score & Positions of GeneSet Members on the Rank Ordered List

  

| SYMBOL | TITLE | RANK IN GENE LIST | RANK METRIC SCORE | RUNNING ES | CORE ENRICHMENT || 1 | HLA-DQA1 | na | 299 | 0.625 | 0.0528 | Yes |
| 2 | FYN | na | 470 | 0.560 | 0.1029 | Yes |
| 3 | HLA-DPB1 | na | 612 | 0.518 | 0.1497 | Yes |
| 4 | CD40 | na | 1048 | 0.440 | 0.1805 | Yes |
| 5 | HLA-DQA2 | na | 1082 | 0.435 | 0.2223 | Yes |
| 6 | CAV1 | na | 1207 | 0.419 | 0.2599 | Yes |
| 7 | MYH8 | na | 1589 | 0.378 | 0.2861 | Yes |
| 8 | SGCB | na | 1611 | 0.376 | 0.3224 | Yes |
| 9 | ICAM1 | na | 2463 | 0.312 | 0.3285 | Yes |
| 10 | PRF1 | na | 2774 | 0.294 | 0.3484 | Yes |
| 11 | CD40LG | na | 3305 | 0.265 | 0.3591 | Yes |
| 12 | HLA-DMB | na | 3372 | 0.261 | 0.3829 | Yes |
| 13 | CD80 | na | 3860 | 0.242 | 0.3926 | Yes |
| 14 | HLA-DRA | na | 3924 | 0.239 | 0.4143 | Yes |
| 15 | MYH4 | na | 4076 | 0.234 | 0.4329 | Yes |
| 16 | HLA-DQB1 | na | 4600 | 0.215 | 0.4389 | Yes |
| 17 | HLA-DPA1 | na | 4808 | 0.207 | 0.4533 | Yes |
| 18 | SGCA | na | 5781 | 0.177 | 0.4426 | No |
| 19 | MYH7B | na | 6460 | 0.158 | 0.4386 | No |
| 20 | MYH6 | na | 7367 | 0.134 | 0.4256 | No |
| 21 | HLA-DRB4 | na | 7427 | 0.132 | 0.4369 | No |
| 22 | HLA-DRB5 | na | 8559 | 0.107 | 0.4148 | No |
| 23 | HLA-DRB1 | na | 9300 | 0.094 | 0.4027 | No |
| 24 | HLA-DOB | na | 10476 | 0.074 | 0.3760 | No |
| 25 | MYH10 | na | 11002 | 0.066 | 0.3674 | No |
| 26 | HLA-DRB3 | na | 11175 | 0.063 | 0.3686 | No |
| 27 | MYH7 | na | 11734 | 0.056 | 0.3580 | No |
| 28 | SGCD | na | 11879 | 0.054 | 0.3592 | No |
| 29 | EIF4G1 | na | 13145 | 0.039 | 0.3265 | No |
| 30 | HLA-G | na | 13698 | 0.033 | 0.3139 | No |
| 31 | CD55 | na | 13872 | 0.032 | 0.3120 | No |
| 32 | RAC2 | na | 13916 | 0.031 | 0.3139 | No |
| 33 | MYH9 | na | 13965 | 0.031 | 0.3155 | No |
| 34 | CXADR | na | 13983 | 0.031 | 0.3180 | No |
| 35 | CASP9 | na | 14196 | 0.029 | 0.3147 | No |
| 36 | HLA-DMA | na | 14540 | 0.025 | 0.3073 | No |
| 37 | MYH2 | na | 14556 | 0.025 | 0.3094 | No |
| 38 | HLA-A | na | 15242 | 0.019 | 0.2915 | No |
| 39 | HLA-C | na | 15354 | 0.018 | 0.2900 | No |
| 40 | HLA-DOA | na | 15373 | 0.018 | 0.2913 | No |
| 41 | CYCS | na | 16260 | 0.011 | 0.2667 | No |
| 42 | EIF4G2 | na | 16294 | 0.010 | 0.2668 | No |
| 43 | RAC1 | na | 16331 | 0.010 | 0.2668 | No |
| 44 | ABL1 | na | 16492 | 0.009 | 0.2630 | No |
| 45 | MYH11 | na | 16745 | 0.007 | 0.2564 | No |
| 46 | ACTG1 | na | 16994 | 0.005 | 0.2497 | No |
| 47 | MYH13 | na | 17626 | -0.001 | 0.2316 | No |
| 48 | HLA-E | na | 18198 | -0.006 | 0.2157 | No |
| 49 | ABL2 | na | 18810 | -0.011 | 0.1991 | No |
| 50 | HLA-F | na | 19312 | -0.016 | 0.1862 | No |
| 51 | ITGB2 | na | 20232 | -0.024 | 0.1621 | No |
| 52 | BID | na | 20508 | -0.027 | 0.1568 | No |
| 53 | CCND1 | na | 20546 | -0.027 | 0.1584 | No |
| 54 | ACTB | na | 22412 | -0.047 | 0.1092 | No |
| 55 | MYH14 | na | 22684 | -0.051 | 0.1064 | No |
| 56 | DMD | na | 23011 | -0.055 | 0.1024 | No |
| 57 | HLA-B | na | 23071 | -0.056 | 0.1061 | No |
| 58 | CASP8 | na | 23310 | -0.059 | 0.1051 | No |
| 59 | CD28 | na | 24062 | -0.071 | 0.0903 | No |
| 60 | LAMA2 | na | 24087 | -0.071 | 0.0966 | No |
| 61 | CASP3 | na | 24828 | -0.083 | 0.0834 | No |
| 62 | MYH3 | na | 25442 | -0.093 | 0.0748 | No |
| 63 | EIF4G3 | na | 26582 | -0.117 | 0.0534 | No |
| 64 | ITGAL | na | 27827 | -0.146 | 0.0319 | No |
| 65 | MYH15 | na | 30036 | -0.212 | -0.0110 | No |
| 66 | DAG1 | na | 30263 | -0.220 | 0.0041 | No |
| 67 | SGCG | na | 30268 | -0.220 | 0.0257 | No |
| 68 | MYH1 | na | 31917 | -0.289 | 0.0065 | No |
| 69 | RAC3 | na | 32406 | -0.320 | 0.0239 | No |
| 70 | CD86 | na | 33693 | -0.439 | 0.0299 | No |
Table: GSEA details [plain text format]

  

Fig 2: KEGG\_VIRAL\_MYOCARDITIS      
 Blue-Pink O' Gram in the Space of the Analyzed GeneSet

  

Fig 3: KEGG\_VIRAL\_MYOCARDITIS: Random ES distribution      
 Gene set null distribution of ES for **KEGG\_VIRAL\_MYOCARDITIS**
